# Assessment of three fasting plasma glucose targets for insulin glargine-based therapy in people with type 2 diabetes mellitus in China: study protocol for a randomized controlled trial

**DOI:** 10.1186/s13063-016-1588-6

**Published:** 2016-09-26

**Authors:** Wenying Yang, Zhaojun Yang, Jing Zhao, Hai Lu, Tianhong Luo

**Affiliations:** 1 China–Japan Friendship Hospital, East Yinghuayuan Street, Hepingli, Beijing, 100029 People’s Republic of China; 2 Jing’An Kerry Centre, 19F, Tower III, No.1228M, Yan’an Road, Shanghai, 200040 People’s Republic of China

**Keywords:** Type 2 diabetes mellitus, Oral antidiabetic drugs, Fasting plasma glucose, Self-monitor fasting blood glucose, glycated hemoglobin

## Abstract

**Background:**

A large proportion of patients with T2DM in China do not meet accepted HbA1c targets despite the availability of guidelines that describe a treatment pathway for achieving glycemic control. The aim of this study is to identify the fasting plasma glucose (FPG) target that will provide the highest control rate of HbA1c <7 % in Chinese patients with T2DM treated with an insulin glargine-based regimen as an adjunct to an established OAD regimen. This information will support improvements in diabetes care management in China.

**Methods:**

Approximately 934 men and women aged ≥18 to ≤65 years with poorly controlled T2DM will be enrolled and randomized to one of three FPG target groups; ≤5.6 mmol/L, ≤6.1 mmol/L, or ≤7.0 mmol/L. They will be initiated on daily insulin glargine (Lantus®) in addition to their usual OAD regimen for 24 weeks. Patients will self-monitor fasting blood glucose (SM-FBG), and the study physician will titrate the basal insulin dose according to the SM-FBG results. In addition, HbA1c and safety will be recorded. We plan to statistically derive the optimal FPG target for an HbA1c of <7 %.

**Discussion:**

In China, treatment strategies that would achieve an optimum balance between glycemic control (as per HbA1c) and hypoglycemia are imperative to ensure improvements in the management of T2DM. Furthermore, elucidating the contribution of FPG to HbA1c in Chinese patients with T2DM and identifying a predictable relationship between FPG and HbA1c would be a valuable tool for patient self-management of diabetes.

**Trial registration:**

NCT02545842. Registered on 8 September 2015.

## Background

Globally, type 2 diabetes mellitus (T2DM) has become a considerable economic burden owing to the impact of the disease in terms of costs to society, health systems, individuals, and employers, and in terms of a reduction in the productive workforce and productivity in general [1, 2]. In China, the impact of T2DM is particularly disconcerting: the Chinese T2DM population is nearing 100 million individuals—about a quarter of the global T2DM population—and is projected to reach 142.7 million by 2035 [3–5]. Moreover, recent data suggest that, in general, patients with T2DM in China have poor glycemic control; a large observational study conducted at 209 hospitals across China found that patients had a mean hemoglobin A1c (HbA1c) level of 9.6 ± 2.0 % before initiation of basal insulin [6], and it has been reported that only 35.9 % of Chinese T2DM patients who are treated exclusively with oral antidiabetic drugs (OADs) achieve HbA1c levels of <7 % [7].

HbA1c has become the standard for assessing and monitoring glycemic control in patients with diabetes, and HbA1c has been the independent variable against which rates of complications in all major trials have been assessed [8–11]. Several large epidemiological studies have implicated the association of high HbA1c values (i.e., values higher than 7 %) and the development of complications of diabetes, especially atherosclerosis and other microvascular and macrovascular complications [12–14]. Correspondingly, there is a strong association between a decrease in HbA1c and a reduction in T2DM-related complications. For instance, the UK Prospective Diabetes Study (UKPDS) study found a 35 % reduction in the risk of microvascular complications for each 1 % decrement in HbA1c [15]. Furthermore, there is thought to be a relationship between HbA1c and fasting plasma glucose (FPG). To date, several studies have explored the exact nature of this relationship, i.e., the FPG target that would correspond with HbA1c values that define glycemic control. The ORIGIN study demonstrated a graded relationship between FPG ≥5.6 mmol/L and HbA1c levels [16], and a US-based study reported that an FPG target of 5.6–6.1 mmol/L provided an optimum balance between HbA1c and hypoglycemic events [17].

T2DM patients who do not meet HbA1c targets with lifestyle modifications and OADs are commonly initiated on basal insulin as an adjunct to OADs. The safety and efficacy of basal insulin as an adjunct to OADs have been confirmed by several global studies (reviewed by Cahn et al. [18]), and this is a conventional treatment approach to glycemic control and an established practice, especially in Western countries [19]. Nonetheless, there are some limitations to this therapeutic approach; for instance, the TITRATE study showed that although the majority of T2DM patients who were initiated on once-daily basal insulin following inadequate glycemic control with OADs, achieved an HbA1c level of <7 %, they did not achieve FPG targets of 3.9–5.0 mmol/L or 4.4–6.1 mmol/L [20]. Furthermore, there are profound differences in T2DM pathophysiology in Asians and Westerns [21], and the FPG targets for basal insulin therapy in Caucasian patients may well not be appropriate for Asians. In Asians, T2DM develops at a lower mean body mass index than in individuals of European descent, and Asian T2DM is characterized by early β cell dysfunction, which may necessitate early initiation of insulin therapy. In general, Asian patients with T2DM have a higher degree of insulin resistance, higher postprandial glucose excursions, and higher incidence of hypoglycemia than Westerners with T2DM [22, 23].

Currently, the relationship between HbA1c and FPG in Chinese patients with T2DM is poorly understood. The Chinese Diabetic Society’s treatment guidelines recommend the initiation of insulin (basal or premix insulin) if lifestyle changes and an OAD regimen fail to achieve glycemic control [24]. However, only a limited number of studies have investigated the efficacy of insulin glargine-based treatment in Chinese patients with T2DM to date. Moreover, these studies were unable to identify a clear and consistent FPG target that would suggest appropriate glycemic control [25, 26]. Data are needed to elucidate the contribution of FPG to HbA1c in Chinese patients with T2DM. Identifying effective FPG targets would enable Chinese physicians to optimally treat their patients with reduced concerns about hypoglycemia. Also, finding a predictable relationship between FPG and HbA1c could be a valuable tool in patient self-management of diabetes, as FPG can be self-monitored.

The primary objective of the present study is to identify the FPG target that will provide the highest control rate of HbA1c <7 % in Chinese patients with T2DM treated with an insulin glargine (Lantus®)-based regimen as an adjunct to an established regimen of OADs. To this end, the study’s primary end point is HbA1c ≤7 % after 24 weeks of treatment with insulin glargine in addition to OADs ± sulfonylurea (SU).

Secondary end points include: the control rate of HbA1c ≤6.5 % and HbA1c <7.0 % in patients who achieve their assigned FPG target; the percentage of patients who achieve HbA1c <7 % without hypoglycemia per FPG target group; reduction in HbA1c, FPG and postprandial glucose (PPG) from baseline (week 1) to week 24; and the mean insulin dose of each FPG group at the end of the treatment period. Safety and quality of life will also be assessed. For safety end points, frequency and severity of adverse events, rate of hypoglycemia, subgroup analysis of hypoglycemia occurrence by the use of SU intra-group and inter-group, change of laboratory tests and vital signs, change in weight in each treatment arm from beginning to the end of the study will be assessed.

Our study will also conduct a cost-effectiveness analysis evaluating patient quality of life and medical costs to better understand the impact of improved HbA1c control on patients’ quality of life and financial burden.

## Methods

This prospective, randomized, three-arm parallel-group, open-label, treat-to-target study will be conducted at multiple centers across China (clinical study number: NCT02545842). The study will be executed in accordance with the Declaration of Helsinki and in line with the principles of Good Clinical Practice.

### Patients

The study plans to enroll 934 men and women ≥18 to ≤65 years of age with poorly controlled T2DM. Poorly controlled T2DM is defined as an HbA1c of >7 % despite stable treatment with one to three OADs for a period of at least 3 months prior to study entry. Patients with HbA1c ≤10.5 % will not be eligible for enrollment.

### Study design

**Fig. 1:**
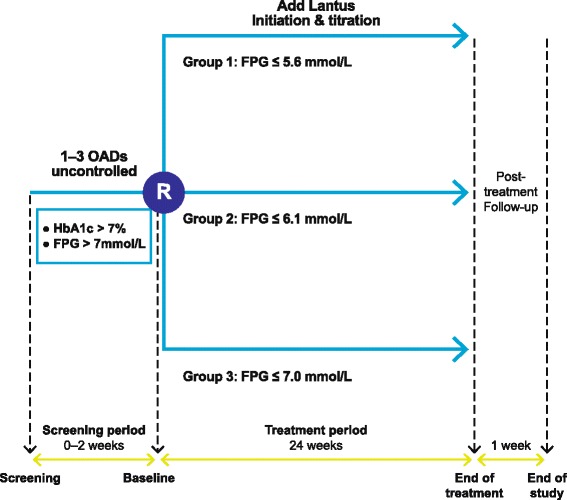
Schematic flow diagram of the study design. *FPG* fasting plasma glucose, *OAD* oral antihyperglycemic drug

The study design is shown in Fig. 1. Briefly, patients will be stratified according to the presence/absence of SU in their usual treatment regimen and randomized in a 1:3:3 ratio to one of three FPG target groups: ≤5.6 mmol/L, ≤6.1 mmol/L, or ≤7.0 mmol/L.

Patients will be required to continue with their usual OADs for the duration of the study. Changes to a patient’s usual treatment can be made at the investigators’ discretion based on safety reasons (i.e., hypoglycemia) and in accordance with Chinese treatment guidelines and local label indications. However, patients will not be allowed to discontinue or initiate SU during the treatment period.

All study patients will initiate insulin glargine at a dose of 0.2 U/kg as an adjunct therapy to their usual treatment regimen. Patients will be required to inject insulin glargine using a prefilled disposable pen, which contains a 3-mL cartridge of insulin glargine suspension for injection (Lantus® SoloSTAR®, Sanofi-Aventis Deutschland GmbH, Frankfurt, Germany).

**Fig. 2:**
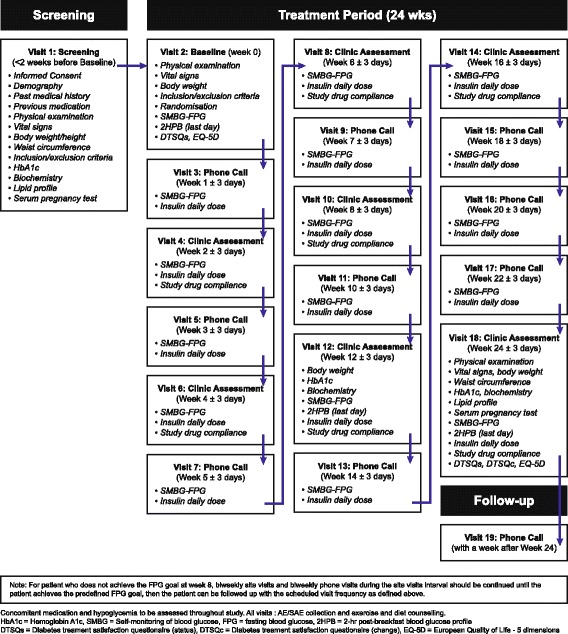
Schedule of visits, treatment, and data collection

During the 24-week treatment period, patients will self-monitor fasting blood glucose (SM-FBG) at least three times per week for the first 8 weeks and twice per week from week 8 onward after a fasting period of 8 hours using a provided glucose meter. See Fig. 2 for a schedule of visits and assessments.

The study physician will review the SM-FBG values once a week for the first 8 weeks and every 2 weeks thereafter. For these assessments, patients will be contacted by telephone, and they will visit the clinic on alternate weeks. Also, patients will be required to provide the study physician with three SM-FBG results from three consecutive days before the assessment call or visit as well as the insulin dose administered on the day prior to each visit.

The study physician will titrate the basal insulin dose according to the SM-FBG results and the treatment group to which a patient has been assigned. Table 1 details the titration regimes.

**Table 1:** Insulin dose adjustment for each study group

*FPG (mmol/L)*	*Insulin dose*
All groups ≤ 3.9 or nocturnal hypoglycemia	- 2 U
Group 1: 3.9 < FPG ≤ 5.6	No change
Group 2: 3.9 < FPG ≤ 6.1	
Group 3: 3.9 < FPG ≤ 7.0	
Group 1: FPG > 5.6	+2 U
Group 2: FPG > 6.1	
Group 3: FPG > 7.0	

*FPG* fasting plasma glucose

**Fig. 3:**
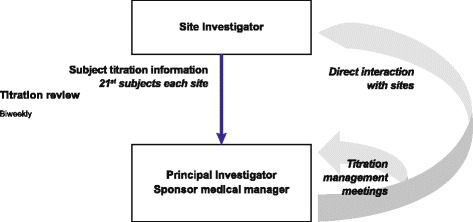
Titration Committee detailed information

To monitor the titration practice and to ensure that appropriate titration algorithms are followed by study physicians at the different study sites, a Study Titration Committee will periodically review the insulin doses that are prescribed. The Committee will convene regularly and, if necessary, will contact sites directly to address or clarify issues in the titration scheme implementation (Fig. 3).

The HbA1c levels of all patients will be measured during the screening visit (baseline), which will take place within 2 weeks before study entry, and then after 12 and 24 weeks of treatment. A 2-h postbreakfast blood glucose profile will be obtained on the day prior to the start of treatment (baseline), at week 12 and 24.

Laboratory tests will be performed on the day of screening, at week 12 and 24. Laboratory data will consist of blood analyses (biochemistry and blood count). Vital signs, including blood pressure and heart rate, will be measured on the day of screening, at baseline and week 24. Body weight will be measured on the day of screening, at baseline, at week 12 and 24. The same weighing scale will be used through the study.

### Statistical methods

As per the randomization schedule, 120 patients will be assigned to the FPG ≤5.6 mmol/L group, and 360 patients will be assigned to the FPG ≤6.1 mmol/L and to ≤7.0 mmol/L groups, respectively. After accounting for a dropout rate of approximately 10 %, the study has 85 % power to detect between-group differences of 15 % (45 % versus 30 %) at a two-sided significant level of 0.05. Also, the study has 80 % power to detect differences of 10 % (40 % versus 30 %) between the FPG ≤6.1 mmol/L and ≤7.0 mmol/L groups. Descriptive analysis will be performed for interim analysis of safety and efficacy.

#### Interim analysis

For the interim analysis, a descriptive efficacy and safety analysis will be performed when 300 subjects complete the study.

#### Final analysis

The primary efficacy end point is the percentage of patients reaching HbA1c <7 %, and two hierarchical null hypotheses (H_1_ and H_2_) are defined to identify the optimal FPG target for an HbA1c of <7 %.

H_1_ presumes no difference between the FPG <5.6 mmol/L and the FPG <7.0 mmol/L target groups, and H_2_ presumes no difference between the FPG <6.1 mmol/L and FPG <7.0 mmol/L target groups. These hypotheses will be tested in sequence, if need be, i.e., H_2_ will only be tested if H_1_ is rejected.

A subgroup analysis of control rate of HbA1c <7 % by duration of diabetes, duration of OAD treatment, baseline FPG, baseline HbA1c, and age will also be conducted.

Changes from baseline HbA1c levels, FPG, and PPG will be estimated as: change = value at postbaseline visit – value at baseline visit.

Changes from baseline to each postbaseline visit will be estimated using a mixed model [PROC MIXED in SAS (SAS Institute, Cary, NC, USA) or similar], with the results used to test whether there are any differences in the change from baseline by treatment arm. The model will use the end-of-study HbA1c, FPG, or PPG value as the dependent variable, with treatment, stratum, and study site as fixed effects, baseline HbA1c, FPG, or PPG as a covariate, and patient/visit as a repeated measure indicator. Treatment-emergent adverse events, including episodes of hypoglycemia as identified from patient diaries, will be summarized. The number of events, and the number and percentage of patients experiencing hypoglycemia, including symptomatic hypoglycemia, confirmed hypoglycemia, severe hypoglycemia, and nocturnal hypoglycemia, will be compared between FPG target groups and between patients stratified according to the use of SU (with or without SU).

## Discussion

Effective diabetes care management requires practical implementation of evidence-based treatment strategies in routine-care settings. Although Chinese physicians are familiar with Chinese Diabetes Society guideline recommendations for the management of T2DM, evidence shows that many Chinese T2DM patients have poor glycemic control [6, 27, 28]. A possible reason for this is the overly cautious approach to insulin initiation and titration, which, in turn, is thought to be a result of physician anxiety about invoking events of hypoglycemia in patients.

Although hypoglycemia can be the result of tight glycemic control, the consequences of uncontrolled blood glucose levels are debilitating and irreversible.

The current gold standard for monitoring glycemic control in T2DM is HbA1c levels, with HbA1c ≤6.5 or <7 % being considered the optimum. HbA1c levels are tested in the laboratory and usually every 3 months, and so this procedure is not available to patients as a self-monitoring tool [9–11]. Several studies have tried to identify a relationship between HbA1c and FPG, but overall the results are inconclusive [16, 17].

Knowledge of the relationship between HbA1c and FPG would allow for patient self-monitoring of glycemic control because FPG would serve as a proxy for HbA1c. Moreover, it would allow for treatment strategies that achieve an optimum balance between glycemic control (as per HbA1c) and hypoglycemia.

The primary objective of BEYOND III is to identify the FPG target that would provide the highest control rate for HbA1c with low rates of hypoglycemia using insulin glargine as an adjunct treatment to OADs in Chinese patients with T2DM. These results would inform about the best practice for diabetes care management in China. Furthermore, the study will assess the cost-effectiveness of the insulin glargine treatment regimen so as to contribute to current pharmacoeconomic data. A recent meta-analysis reported that the economic impact of T2DM is considerable, but more data are needed from developing economies such as China and India where the healthcare cost of T2DM is expected to soar because of the size of the T2DM population in these countries [29].

For BEYOND III, three FPG targets were identified: ≤5.6 mmol/L, ≤6.1 mmol/L, and ≤7 mmol/L. The lower targets are based on the recommendations from international and domestic guidelines, and previous study results [9, 10, 26], whereas the 7.0 mmol/L target is within the range recommended by 2014 Chinese diabetes guidelines (4.4–7.0 mmol/L) [24] and widely accepted by Chinese physicians.

## Trial status

This study is currently recruiting participants.

## Abbreviations

FPG: fasting plasma glucose
HbA1c: glycated hemoglobin
OAD: oral antidiabetic drug
PPG: postprandial glucose
SM-FBG: self-monitoring fasting blood glucose
SU: sulfonylurea
T2DM: type 2 diabetes mellitus
UKPDS: UK Prospective Diabetes Study
